# Comparative Quality Assessment of Artificial Intelligence in Patient Education on Platelet-Rich Plasma (PRP) Therapy

**DOI:** 10.3390/jpm16030173

**Published:** 2026-03-23

**Authors:** Jonas Krueckel, Dominik Szymski, Nura Ahmad, David Schiffelholz, Johannes Weber, Siska Buchhorn, Tomas Buchhorn, Kai Fehske, Siegmund Lang, Volker Alt, Franz Hilber

**Affiliations:** 1 Department of Trauma Surgery, University Medical Centre Regensburg, 93053 Regensburg, Germany; 2 Department of Plastic, Hand and Reconstructive Surgery, University Medical Centre Regensburg, 93053 Regensburg, Germany; 3 Department of Orthopaedic, Trauma and Rehabilitative Medicine, Musculoskeletal University Center Munich, Ludwig-Maximilians-University (LMU), 81377 Munich, Germany; 4 Sporthopaedicum Regensburg, 93059 Regensburg, Germany; 5 Department of Orthopedic and Trauma Surgery, Johanniter Waldkrankenhaus Bonn, 53177 Bonn, Germany; 6 Department of Trauma, Hand, Plastic und Reconstructive Surgery, University Hospital Wuerzburg, 97080 Wuerzburg, Germany

**Keywords:** platelet-rich plasma, PRP, artificial intelligence, large language models, sports medicine, patient education

## Abstract

**Background**: Platelet-rich plasma (PRP) therapy is increasingly used for musculoskeletal conditions, yet patients seeking supplementary information online encounter resources of variable quality. Large language models (LLMs) such as ChatGPT and Google Gemini may support patient education, but their performance in answering common patient questions about PRP therapy has not been well characterized. **Methods**: This study compared the quality of responses generated by ChatGPT-4, ChatGPT-3.5, and Google Gemini to common PRP-related patient questions. Ten frequently asked PRP-related questions were identified through a structured search of online sources, PubMed, Google Trends, and AI-assisted query generation. Each question was submitted to the three LLMs using a standardized prompt designed to elicit clear and empathetic responses. Five orthopedic surgeons, blinded to model identity, assessed each answer using a previously published four-tier rating framework. Secondary metrics included exhaustiveness, clarity, empathy, and response length. **Results**: All models produced mostly satisfactory answers. ChatGPT-3.5 received the highest proportion of excellent ratings (70%), compared with 40% for ChatGPT-4 and 22% for Gemini, and outperformed both models in overall quality. The most common limitation across models was insufficient detail. ChatGPT-4 and Gemini performed similarly in several categories, although Gemini was rated lower in empathy and comprehensiveness. Overall differences between models were statistically significant. **Conclusions**: Commonly available LLMs were able to provide mostly satisfactory responses to patient questions about PRP. However, important limitations remained, particularly with respect to detail and individualization. These tools may support initial patient information-seeking, but they should complement rather than replace expert medical counseling.

## 1. Introduction

Platelet-rich plasma (PRP) therapy has gained considerable traction in recent years as a regenerative, patient-specific treatment option for a wide range of musculoskeletal conditions, including osteoarthritis, tendinopathies, and degenerative disorders of the lumbar spine [1,2,3]. In the context of both joint replacement and lumbar spine surgery, there is growing emphasis on exhaustively utilizing conservative treatment modalities before proceeding to invasive surgical interventions such as arthroplasty or spinal fusion [4,5]. PRP has therefore attracted increasing interest as part of individualized, non-operative treatment strategies aimed at symptom relief, functional improvement, and potential delay or avoidance of surgery, despite a heterogeneous evidence base that limits uniform recommendations and may contribute to patient uncertainty [6].

In addition to orthopedic indications, PRP is also used in other fields such as plastic and aesthetic surgery, including thumb carpometacarpal joint osteoarthritis, wrinkle treatment, and stimulation of hair growth [7,8]. As interest in PRP continues to grow, so too does the demand for clear, accurate, and accessible information. Many patients actively seek to better understand this evolving therapy, often turning to online sources to supplement information provided during clinical consultations [9]. However, studies have highlighted concerns regarding the quality and readability of such online resources [10,11].

At the same time, large language models (LLMs) such as ChatGPT have emerged as powerful tools capable of delivering medical information on demand. By generating individualized responses to health-related questions, these models offer a novel avenue for patient education [12,13,14,15,16]. However, despite their accessibility and speed, concerns persist regarding the accuracy, depth, and clarity of the content they provide [17,18,19].

Inconsistent response quality and limited source transparency may contribute to misunderstanding or unrealistic expectations, particularly in PRP therapy, where expert consensus is only partial and important aspects of clinical application remain insufficiently standardized [20]. High-quality educational materials are critical for fostering informed decision-making. Accurate and empathetic communication not only supports patient autonomy but also strengthens trust in the therapeutic process [21,22,23]. As healthcare continues to integrate digital technologies, LLMs have the potential to serve as valuable adjuncts in the patient–provider relationship, provided that the information they generate meets standards of medical reliability and readability [24,25].

Despite their promise, key questions remain regarding whether LLMs can provide medically sound, understandable, and context-appropriate answers to patient questions about PRP therapy. This study therefore compared the quality of responses generated by ChatGPT-3.5, ChatGPT-4, and Google Gemini to common PRP-related queries, as evaluated by orthopedic reviewers.

## 2. Materials and Methods

### 2.1. Design and Data Assessment

**Figure 1:**
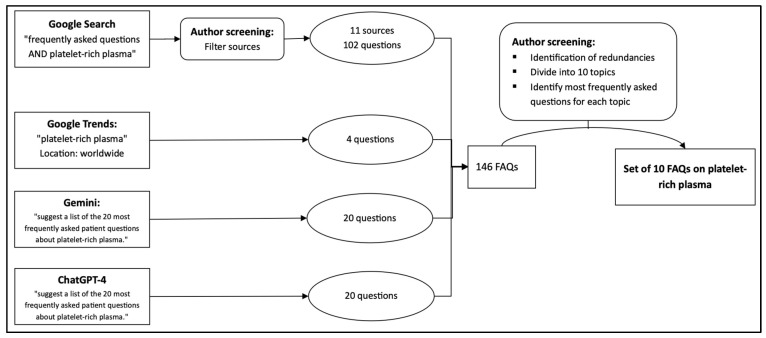
Selection Process for Identifying the Top 10 Frequently Asked Questions About PRP Therapy.

To systematically identify the most relevant and frequently asked patient questions regarding PRP therapy, a multi-layered search strategy was implemented. Google and PubMed searches were performed on 29 March 2024 in Regensburg, Germany, using English-language search settings and Google Chrome in incognito mode to reduce personalization effects. The Google query used was “frequently asked questions AND platelet rich plasma OR PRP”. From the Google results, the first 20 hits were screened for eligibility according to predefined criteria. Eligible sources had to be written in English and structured in a question-and-answer (FAQ) format. Sources were excluded if they focused exclusively on highly specific provider-dependent content or on non-generalizable applications. Because publication dates were not consistently available for all web-based FAQ pages, the date of publication was not used as a strict exclusion criterion for website screening. To supplement the literature and broaden the scope, ChatGPT-4 was queried using the prompt: “suggest a list of the 20 most frequently asked patient questions about platelet-rich plasma.” In addition, search trends from Google Trends were analyzed using the keyword “platelet-rich plasma” to identify high-interest topics that reflect current patient concerns. The web-based screening and complementary database/trend search resulted in a set of 146 unique candidate questions. The websites contributing questions to the initial pool are listed in Supplementary Table S1. Of the first 20 Google results screened, 11 websites met the eligibility criteria and contributed questions to the final candidate pool. The 146 candidate questions were first assigned to 10 thematic categories representing distinct aspects of PRP therapy. Within each category, questions with identical or closely similar meaning were grouped together, and their frequency of occurrence across sources was recorded. For each thematic category, the most frequently recurring question was then selected for inclusion in the final set. This reduction process yielded 10 representative FAQs, with each question corresponding to one predefined thematic category and collectively covering clinical, procedural, and outcome-related aspects of PRP therapy (Table 1). The screening and thematic grouping were performed by a single reviewer. A corresponding flowchart outlining the selection and refinement process is illustrated in Figure 1. For example, questions such as “How long does PRP take to work?” and “When will I notice improvement after PRP?” were considered conceptually overlapping and grouped within the same category.

To assess the capabilities of large language models in providing patient education on PRP therapy, ChatGPT-4, ChatGPT-3.5, and Google Gemini were queried via their publicly accessible web interfaces using the free standard versions available at the time of data collection. Queries were performed in April 2024 in incognito mode, without user-specific customization or additional system instructions. The same standardized prompt was used verbatim for all models, with only the respective patient question inserted at the end:

“Act as a doctor and expert in the field of use of platelet-rich plasma (PRP) in an orthopedic/surgical setting, who is up to date with the latest scientific research and has years of experience counseling patients with empathy and clarity. Provide a comprehensive and easily understandable answer to the following question about platelet-rich plasma (PRP)! Limit your answer to 150 words and focus on the most important aspects to ensure patient information: (…)”.

To avoid carryover effects, each query was entered in a new chat. No adjustable generation settings, such as temperature, were modified through the web-based interfaces. All responses remained within the predefined 150-word limit.

ChatGPT-3.5 and ChatGPT-4 are large language models developed by OpenAI, while Google Gemini is developed by Google. These systems are designed to generate natural-language responses to user prompts and may differ in response style, detail, and content [26]. The responses generated by the large language models were evaluated by five board-certified orthopedic surgeons, all of whom had clinical experience in the use of PRP therapy. The evaluators were blinded to the identity of the specific model that generated each response.

A previously published four-tier evaluation framework was employed to assess the quality and utility of each response [27]. The reviewers classified each answer into one of four categories. An “excellent” response was defined as fully accurate, complete, and requiring no clarification; a “satisfactory response requiring minimal clarification” was generally correct but contained minor omissions or slight simplifications; a “satisfactory response requiring moderate clarification” was found to be informative but included outdated elements, notable gaps, or lacked contextual nuance; and an “unsatisfactory response” was characterized by significant inaccuracies, vague or generic wording, or misleading content that could result in patient misunderstanding.

For all responses that were not rated as excellent, reviewers were asked to identify the reason for the lower rating by selecting from a set of predefined categories. These included issues such as being off-topic, containing factual errors, including too much or too little information, using unclear or inappropriate language, or presenting other relevant problems. In addition to this qualitative classification, each rater completed a supplemental evaluation comprising four items rated on a five-point Likert scale. These items assessed the perceived exhaustiveness of the response, its clarity, the appropriateness of its length, and the degree to which the response addressed the patient’s concerns with empathy and sensitivity.

### 2.2. Statistical Analysis

Statistical analysis was conducted using GraphPad Prism (version 10.1, GraphPad Software Inc., San Diego, CA, USA). The four-category overall response rating and the five-point Likert-scale secondary ratings were analyzed as ordinal data.

To compare the overall performance ratings of ChatGPT-4, ChatGPT-3.5, and Google Gemini, a Friedman test for repeated measures was applied using matched rater-question combinations across the three models. In this framework, the same reviewer evaluated the three model responses to the same question under identical conditions, yielding 50 matched observations in total (10 questions × 5 reviewers). For the additional Likert-scale outcomes, including exhaustiveness, clarity, empathy, and appropriateness of response length, Friedman tests for repeated measures were likewise performed separately for each dimension using the same matched structure, followed by Dunn’s post hoc tests with Bonferroni adjustment where applicable. Inter-rater reliability for the overall 4-category response rating was additionally assessed using Fleiss’ kappa. Statistical significance was defined as *p* < 0.05. Formal Institutional Review Board approval was not required under local institutional regulations, as the study did not involve patients, patient data, or any intervention.

## 3. Results

**Figure 2:**
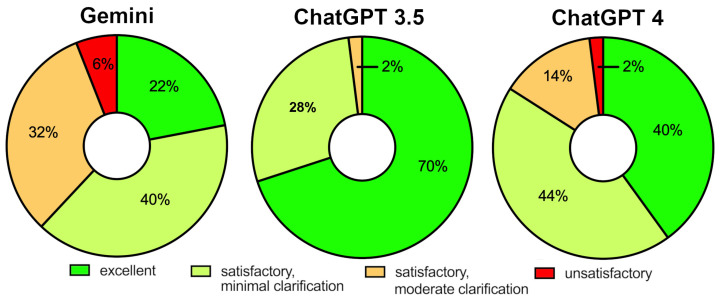
Pie Chart Depicting the Percentage Distribution of Overall Ratings by Orthopedic Surgeons for the Combined Question Set Across All Three LLMs.

The majority of responses across all three language models and the 10 PRP-related questions were judged to be satisfactory, with only a small number deemed unsatisfactory. Descriptive proportions were based on 50 individual ratings per model (10 questions rated by 5 reviewers). On this basis, Google Gemini received an ‘excellent’ rating in 22% of ratings, while 40% were classified as requiring minimal clarification, 32% as requiring moderate clarification, and 6% as unsatisfactory. ChatGPT 3.5 achieved the highest proportion of excellent ratings, with 70% of ratings classified as excellent, 28% as requiring minimal clarification, 2% as requiring moderate clarification, and none as unsatisfactory. ChatGPT 4 received 40% excellent ratings, 44% minimal clarification, 14% moderate clarification, and 2% unsatisfactory ratings. Inter-rater reliability for the overall 4-category response rating was slight (Fleiss’ κ = 0.09). A Friedman test for repeated measures showed a significant difference in overall response quality across models (*p* = 0.0021). Subsequent Dunn’s post hoc tests with Bonferroni adjustment revealed that ChatGPT 3.5 performed significantly better than both Gemini (*p* < 0.001) and ChatGPT 4 (*p* = 0.014). The difference between Gemini and ChatGPT 4, however, did not reach statistical significance (Figure 2).

For all responses that did not receive an “excellent” rating, reviewers were asked to indicate the primary reason for their assessment using predefined categories.

**Figure 3:**
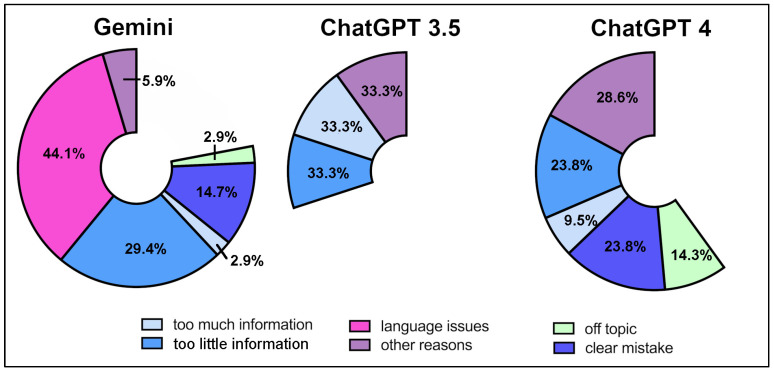
Distribution of Reasons for Non-Excellent Ratings by Orthopedic Surgeons Across Google Gemini, ChatGPT 3.5, and ChatGPT 4, Expressed as Percentages.

Among these sub-excellent responses, the most frequently selected issue for Google Gemini was language problems, cited in 44.1% of cases. This was followed by too little information in 29.4% and clear factual mistakes in 14.7%. Less commonly, off-topic content and too much information were each mentioned in 2.9%, while other reasons accounted for 5.9% of responses. For ChatGPT 3.5, reviewers distributed their assessments evenly among the following categories: too little information, too much information, and other reasons. Each accounted for 33.3% of the non-excellent ratings. No instances of language issues, clear mistakes, or off-topic responses were reported for this model. In the case of ChatGPT 4, the most cited reasons were clear mistakes and too little information, each making up 23.8% of sub-excellent responses. Other reasons were given in 28.6% of cases. Off-topic content was noted in 14.3%, while too much information appeared in 9.5%. Notably, language issues were not reported at all for ChatGPT 4 (Figure 3).

**Figure 4:**
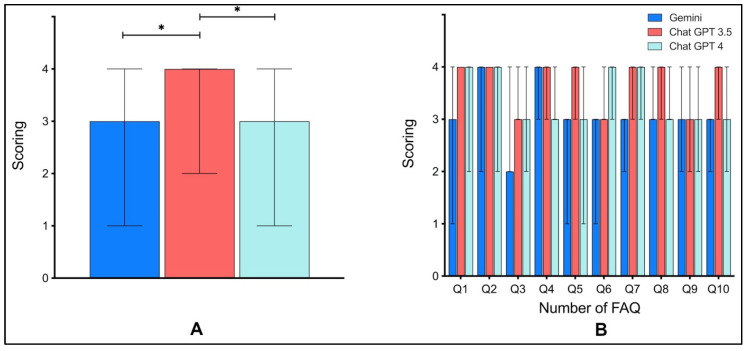
(**A**): Median ratings for the overall quality of responses across Google Gemini, ChatGPT 3.5, and ChatGPT 4. Error bars represent the range (minimum–maximum) of ratings for each model. (**B**): Breakdown of median ratings by individual FAQs, illustrating the performance of each chatbot for specific questions. * = statistically significant.

When aggregating all response ratings, clear differences in overall performance emerged among the three models, with ChatGPT 3.5 receiving notably higher ratings than both ChatGPT 4 and Google Gemini (Figure 4A). However, when assessing the responses question by question, median scores across the 10 PRP-related items were relatively consistent among the models. While minor fluctuations were observed for specific questions, no consistent pattern favoring one model across all items was evident (Figure 4B). Although overall response quality differed between models, differences at the level of individual questions were less pronounced.

**Figure 5:**
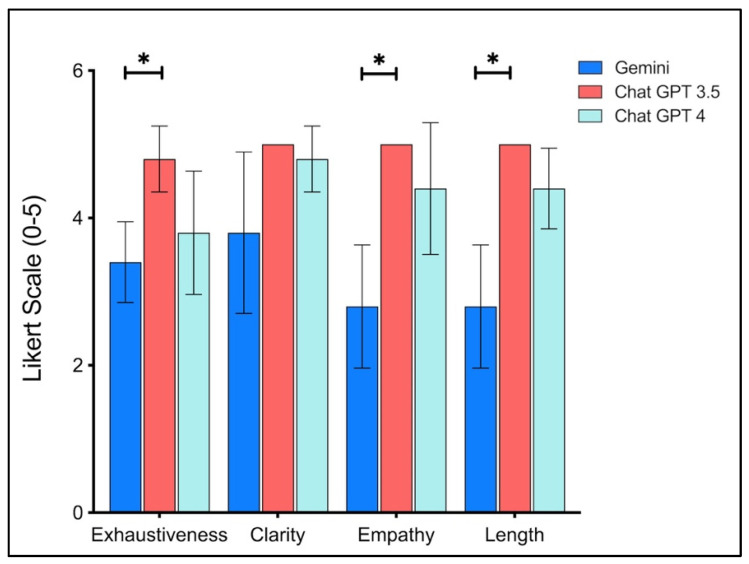
Mean Ratings with Standard Deviations (SD) for Exhaustiveness, Clarity, Empathy/Professionalism, and Response Length Across Google Gemini, ChatGPT 3.5, and ChatGPT 4. * = statistically significant.

The evaluation of exhaustiveness, clarity, empathy and response length revealed notable differences in how the three models were perceived (Figure 5). ChatGPT 3.5 consistently received the highest ratings across all four dimensions, whereas Google Gemini received the lowest ratings overall, and ChatGPT 4 generally showed intermediate values. While both ChatGPT models generally delivered well-rounded and clinically appropriate answers, Google Gemini’s performance appeared less consistent, with limitations in exhaustiveness, empathy and length. The full model-generated responses to all 10 PRP-related questions are provided in Table S2, and question-specific median (IQR) ratings by model are reported in Supplementary Table S3.

## 4. Discussion

The growing capabilities of large language models (LLMs), such as ChatGPT and Google Gemini, are attracting increasing interest in the context of patient education [13,14,15,28,29,30,31,32]. This relevance extends across various medical disciplines, including orthopedics and spine surgery, where patients facing decisions related to spine disorders or joint replacement frequently seek information on conservative and regenerative treatment options prior to invasive surgical interventions. In these contexts, the availability of clear, balanced, and medically accurate content is of particular importance, especially for therapies like PRP, which are often discussed as individualized, non-operative options but are also subject to heterogeneous evidence and variable clinical recommendations.

In this study, we systematically identified commonly asked patient questions about PRP therapy and evaluated the quality of AI-generated responses from ChatGPT 4, ChatGPT 3.5, and Google Gemini, using blinded ratings from orthopedic surgeons.

The findings demonstrate that all three LLMs were capable of generating largely satisfactory responses. ChatGPT 3.5, in particular, achieved the highest proportion of excellent ratings, while ChatGPT 4 and Gemini followed with somewhat lower but still clinically useful outputs. These results indicate that general-purpose LLMs can deliver a solid foundation of information for patients seeking to understand PRP treatment options and expectations. When examining specific aspects of response quality, namely exhaustiveness, clarity, empathy, and response length, ChatGPT 3.5 was generally rated more favorably than the other models. It received the highest median scores across all four categories and showed statistically significant advantages over Google Gemini in clarity, empathy and response length. ChatGPT 4 consistently fell between the two models across all aspects.

These results suggest that while all models were capable of generating useful responses, important differences exist in how they handle tone, completeness, and communicative nuance, highlighting variation not only between models but also across different qualitative facets of patient-oriented communication. At the same time, the slight inter-rater agreement observed in this study (Fleiss’ κ = 0.09) indicates that these comparative differences should be interpreted with appropriate caution. While ChatGPT 3.5 and ChatGPT 4 displayed a more evenly distributed pattern of errors across categories such as off-topic, language issues, and clear mistakes, Gemini’s non-excellent ratings were disproportionately driven by “language issues” (44.1%), indicating a qualitatively distinct limitation in communicative clarity.

It is particularly noteworthy that ChatGPT 3.5, an earlier version of the model, outperformed ChatGPT 4.0 in this specific setting. Several explanations may account for this. One possible factor is that ChatGPT 4, while more powerful in reasoning tasks and abstract comprehension, may generate more verbose or cautious responses that can be perceived as less concise or patient-friendly. In contrast, ChatGPT 3.5 often delivers more direct and structured answers, which may align better with expectations in straightforward medical education contexts. Additionally, differences in fine-tuning objectives between model versions may contribute to variations in tone, confidence, or prioritization of information. While ChatGPT 3.5 achieved the highest proportion of excellent ratings overall (Figure 2), the model also showed greater variability in performance across individual questions (Figure 4B). In contrast, ChatGPT 4 demonstrated a more consistent output pattern, delivering solid, though less exceptional, results across all ten FAQs. This consistency may become particularly relevant in real-world settings, where patients use varying prompts and expect reliable baseline quality regardless of phrasing. In that context, ChatGPT 4’s steadier performance across diverse patient questions may offer an advantage over ChatGPT 3.5’s more variable but occasionally higher-scoring outputs.

Despite the overall strengths observed, common limitations were shared across all models. The most frequently cited reason for suboptimal ratings was a lack of sufficient information. This suggests that even though the models can produce relevant and coherent outputs, they may fall short when dealing with complex, nuanced, or individualized aspects of PRP therapy, particularly in areas requiring precise indication-based guidance or evidence-based recommendations.

An important contextual point is that the questions used in this study covered PRP therapy as a general concept, without restricting the focus to a specific condition such as osteoarthritis or tendinopathy. While this broader scope introduces variability, it reflects the real-world diversity of patient inquiries. PRP is applied across a wide range of conditions, and patients often ask general questions before reaching a disease-specific understanding. Limiting the evaluation to a narrow use case might improve consistency but would reduce generalizability and practical relevance. Therefore, evaluating model performance across a spectrum of commonly asked, generalized PRP questions was considered more appropriate for assessing their utility in patient education.

These observations align with earlier work highlighting the strengths and limitations of LLMs in healthcare communication. Prior studies have shown that AI-generated responses are often rated highly in terms of clarity and tone but may still suffer from factual inaccuracies or oversimplifications [31,32,33,34]. The relatively high frequency of “too little information” ratings in our data echoes previous concerns that current LLMs may underperform when tasked with addressing complex or individualized topics, such as mechanisms of action, indications, or long-term outcomes of PRP therapy [31,35,36].

One factor that may have contributed to the overall high quality of responses in this study was the use of a standardized prompt instructing the models to act as empathetic and knowledgeable medical professionals. This likely improved consistency and tone but also underscores the importance of effective prompt design. As demonstrated in prior work, the phrasing and specificity of the input prompt can strongly influence the output quality of LLMs. Without well-structured prompts, the same models may deliver fragmented, vague, or even misleading responses, highlighting the need for training and oversight in clinical applications of AI [37,38].

### 4.1. Clinical Implications

Given that general-purpose LLMs are trained on broad, uncurated data sources, their content quality reflects the variability of the underlying information. While they can serve as powerful starting points for patient education, more specialized applications, such as institution-specific LLMs trained on clinical protocols, outcomes data, and physician-authored material, may offer substantial advantages in precision and trustworthiness. Custom models tailored to musculoskeletal medicine or regenerative therapies like PRP could be particularly impactful in supporting informed, patient-specific communication.

Within the limits of this exploratory study, the results suggest that LLMs may serve as useful adjuncts in orthopedic patient education, particularly when integrated into a clinician-led workflow. For instance, AI-generated answers could provide preliminary drafts that clinicians refine and personalize, potentially improving efficiency without compromising quality. As prompt engineering techniques continue to evolve and as models become more customizable, the role of LLMs in enhancing patient engagement and communication is likely to expand further.

Future research should assess how patients perceive and respond to AI-generated explanations about PRP, especially when compared to traditional physician-delivered information. In addition, incorporating advanced prompting strategies, such as reasoning chains or context-aware templates, could further enhance model performance for nuanced clinical topics [39]. Understanding how these tools function in real-world settings and how they can be safely and ethically deployed will be essential to maximizing their value in clinical practice.

### 4.2. Limitations

This study has several limitations that should be taken into account. First, the evaluation of responses was performed exclusively by orthopedic surgeons with experience in PRP therapy. While this ensures clinical relevance and content accuracy, it does not reflect the perspective of patients, who may place greater emphasis on accessibility, empathy, or tone. Inter-rater reliability for the overall 4-category response rating was slight (Fleiss’ κ = 0.09). The low Fleiss’ kappa value suggests that some degree of subjectivity remained in the overall categorical assessment despite the use of predefined rating categories. In addition, the effective sample size was limited, as the study was based on 10 questions evaluated by 5 reviewers, which restricts the generalizability of the findings.

Future research should incorporate patient feedback to more fully assess how AI-generated content is perceived and understood by end users.

Second, the study did not compare AI-generated responses to those written by physicians. Including expert-generated answers as a reference standard would allow for a more precise evaluation of the quality and reliability of LLM outputs and help identify meaningful differences or gaps in content and communication style.

Third, while this study focused on general-purpose LLMs that are publicly accessible, medical language models, such as Med-PaLM 2 or ClinicalGPT, were not included. These domain-specific systems may offer more accurate and detailed explanations but are not currently widely available to patients.

Finally, the questions analyzed in this study were intentionally broad and not limited to a single disease indication such as osteoarthritis or tendinopathy. This reflects the real-world diversity of patient inquiries about PRP but may have introduced variability in content expectations. It is also worth noting that LLMs evolve rapidly, and the outputs generated in this study reflect only the specific model versions available at the time of data collection. Future iterations may differ significantly in content, accuracy, and communication style, limiting the generalizability of these results over time. This rapid pace of development poses a challenge for longitudinal evaluation but also highlights the potential for ongoing improvements in AI-assisted patient communication.

## 5. Conclusions

This study demonstrates that commonly available large language models (LLMs) can provide generally satisfactory and comprehensible responses to frequently asked patient questions about platelet-rich plasma (PRP) therapy. Their ability to deliver relevant, clear, and empathetic information highlights their potential to support patient education in the context of regenerative medicine. Nonetheless, important limitations persist, with insufficient detail representing the most frequently identified shortcoming across models, particularly when information requires individualized clinical judgment. While LLMs may serve as useful tools to assist with initial patient engagement and information-seeking, they are not a substitute for medical expertise and should be integrated thoughtfully into broader communication strategies guided by healthcare professionals.

## Figures and Tables

**Table 1:** Overview of Frequently Asked Questions (FAQs) Provided to Large Language Models (LLMs), labeled Q1–Q10.

FAQS
Q1: What is platelet-rich plasma (PRP) therapy?
Q2: How do PRP injections work?
Q3: What conditions can PRP treat?
Q4: How long does it take to recover from a PRP therapy?
Q5: How often should I repeat platelet-rich plasma procedures?
Q6: How effective is PRP treatment?
Q7: What are the side effects associated with PRP injections?
Q8: How painful is a PRP injection?
Q9: How much does PRP therapy cost, and is it covered by insurance?
Q10: How do I know if PRP is the right treatment for me?

## Data Availability

The data presented in this study are available on request from the corresponding author.

## Supplementary Materials

The following supporting information can be downloaded at https://www.mdpi.com/article/10.3390/jpm16030173/s1, Table S1: Web-based FAQ sources contributing to the initial pool of PRP-related patient questions, Table S2: Full Model-Generated Responses to Frequently Asked Questions, Table S3: Question-specific median (IQR) overall ratings by model.

## Abbreviations

The following abbreviations are used in this manuscript:

PRP	Platelet-Rich Plasma
LLMs	Large Language Models
