# Supplementary material for: Comparative Quality Assessment of Artificial Intelligence in Patient Education on Platelet-Rich Plasma (PRP) Therapy
Source: J Pers Med. 2026 Mar 23;16(3):173. doi: 10.3390/jpm16030173 (PMC13028038; doi:10.3390/jpm16030173)
Supplement: Supplementary file 1 [file jpm-16-00173-s001.zip › supplementary_table_S3.pdf]

Supplementary Table S3. Question-specific median (IQR) overall ratings by model

Question	Gemini	ChatGPT-3.5	ChatGPT-4
Q1	3.0 (2.0–3.0)	4.0 (4.0–4.0)	4.0 (3.0–4.0)
Q2	4.0 (3.0–4.0)	4.0 (4.0–4.0)	4.0 (3.0–4.0)
Q3	2.0 (2.0–3.0)	3.0 (3.0–4.0)	3.0 (2.0–3.0)
Q4	4.0 (3.0–4.0)	4.0 (4.0–4.0)	3.0 (3.0–3.0)
Q5	3.0 (2.0–3.0)	4.0 (4.0–4.0)	3.0 (2.0–3.0)
Q6	3.0 (2.0–3.0)	3.0 (3.0–4.0)	4.0 (4.0–4.0)
Q7	3.0 (2.0–3.0)	4.0 (3.0–4.0)	4.0 (4.0–4.0)
Q8	3.0 (3.0–4.0)	4.0 (4.0–4.0)	3.0 (3.0–3.0)
Q9	3.0 (2.0–3.0)	3.0 (3.0–4.0)	3.0 (3.0–3.0)
Q10	3.0 (2.0–3.0)	4.0 (4.0–4.0)	3.0 (3.0–3.0)

Values are shown as median (interquartile range) across five expert raters for each question and model.
