# Supplementary material for: Comparative Quality Assessment of Artificial Intelligence in Patient Education on Platelet-Rich Plasma (PRP) Therapy
Source: J Pers Med. 2026 Mar 23;16(3):173. doi: 10.3390/jpm16030173 (PMC13028038; doi:10.3390/jpm16030173)
Supplement: Supplementary file 1 [file jpm-16-00173-s001.zip › Supplementary_Table_S1_web_sources.pdf]

Supplementary Table S1. Web-based FAQ sources contributing to the initial pool of PRP-related patient questions

No.	Website / Organization	URL
1	Vanguard Spine & Sport	https://vanguardspine.com/blog/the-top-10-frequently-asked-questions-about-prp/
2	Endeavor Health	https://swedishcovenant.org/for-patients-and-visitors/medical-services/chicago-orthopedics/platelet-rich-plasma-(prp)-injection-faqs
3	Advanced Orthopedic & Sports Medicine Institute	https://www.aosmifl.com/plateletrich-plasma-injections-faqs.php
4	Orthopedic Specialists of Seattle	https://orthopedicspecialistsofseattle.com/patient-info/prp-treatment-frequently-asked-questions/
5	Proliance Surgeons	URL not available / not recorded
6	Animas Orthopedic Associates	https://www.animasorthopedics.com/pdfs/aoa-prp-faq.pdf
7	Delo Sports Medicine	https://delosportsmedicine.com/frequently-asked-questions/
8	Reflex Knee Specialists	https://www.reflexknees.com/treatments/platelet-rich-plasma-prp/prp-frequently-asked-questions/
9	Orthopaedic Medical Group of Tampa Bay	https://www.omgtb.com/platelet-rich-plasma-therapy/
10	Desert Spine and Sport Physicians	URL not available / not recorded
11	Michigan Center for Regenerative Medicine	https://regenerativemedicinemichigan.com/faq/
