# Supplementary material for: Comparative Quality Assessment of Artificial Intelligence in Patient Education on Platelet-Rich Plasma (PRP) Therapy
Source: J Pers Med. 2026 Mar 23;16(3):173. doi: 10.3390/jpm16030173 (PMC13028038; doi:10.3390/jpm16030173)
Supplement: Supplementary file 1 [file jpm-16-00173-s001.zip › Supplementary_Table_S2_titlefixed3.pdf]

# Supplementary Table S2.

## Full Model-Generated Responses to Frequently Asked Questions

| **Question:**<br>*"What is platelet-rich plasma (PRP) therapy?"* | excellent answer not requiring clarification | satisfactory, requiring minimal clarification | satisfactory, requiring moderate clarification | unsatisfactory, requiring substantial clarification |
|---|---|---|---|---|
| **ChatGPT-3.5:**<br>Platelet-rich plasma (PRP) therapy involves injecting a concentrated form of your own platelets, rich in growth factors, into damaged tissues to stimulate healing and reduce inflammation. It's a minimally invasive procedure commonly used in orthopedic and surgical settings to treat various conditions like tendon injuries, osteoarthritis, and ligament tears. PRP harnesses your body's natural healing mechanisms, potentially speeding up recovery and reducing reliance on medications or surgery. While research continues to explore its effectiveness, PRP is generally considered safe with minimal risk of adverse effects. However, individual results may vary, and it's crucial to discuss potential benefits and limitations with your healthcare provider. | | | | |
| **ChatGPT-4:**<br>Platelet-rich plasma (PRP) therapy is a treatment that uses a concentration of a patient's own platelets to accelerate the healing of injured tendons, ligaments, muscles, and joints. In this process, a small amount of your blood is drawn and spun in a centrifuge to separate the platelets from other blood components. The concentrated platelet-rich plasma is then injected directly into the injured or diseased body part. The high concentration of platelets delivers an abundance of growth factors that promote natural tissue healing and regeneration. PRP therapy is increasingly used in orthopedic and surgical settings to enhance recovery and reduce pain. While it shows promise, the effectiveness of PRP can vary, and it's important to consult with a healthcare professional to determine if it's the right option for your specific condition. | | | | |
| **Gemini (Google Bard):**<br>As an orthopedic surgeon who uses PRP therapy, let's break it down. PRP stands for Platelet-Rich Plasma. Imagine your blood like a soup – platelets are tiny responders in that soup, containing healing factors. In PRP therapy, we take a small amount of your blood, spin it down to concentrate those platelets, and inject it into your injured area.<br><br>Think of it as a booster shot for your body's own healing process. The concentrated platelets release growth factors that may help speed up healing of tendons, ligaments, and cartilage.<br><br>It's important to know that research is ongoing, and PRP isn't a magic bullet for every injury. But for | | | | |

| | | | | |
|---|---|---|---|---|
| certain conditions, it can be a great option to consider! | | | | |

| **Question:**<br>*"How do PRP injections work?"* | excellent answer not requiring clarification | satisfactory, requiring minimal clarification | satisfactory, requiring moderate clarification | unsatisfactory, requiring substantial clarification |
|---|---|---|---|---|
| **ChatGPT-3.5:**<br>PRP injections work by utilizing the body's own healing mechanisms. Platelets, found in blood, contain growth factors crucial for tissue repair. During a PRP procedure, a small amount of your blood is drawn and centrifuged to separate platelets. The resulting PRP, rich in these growth factors, is injected into the injured area, stimulating tissue regeneration and reducing inflammation. This can accelerate healing in orthopedic and surgical settings, such as tendon injuries, osteoarthritis, and tissue damage. While PRP holds promise, individual responses vary, and it may not be suitable for all conditions. Always consult with your healthcare provider to determine if PRP is right for you. | | | | |
| **ChatGPT-4:**<br>Platelet-rich plasma (PRP) injections are a treatment often used in orthopedics to aid in the healing of injured tendons, ligaments, muscles, and joints. By concentrating platelets, which are cells in your blood that help tissue to heal and grow new cells, PRP injections work by using your own blood to promote healing in damaged areas.<br><br>The process involves drawing a sample of your blood, spinning it in a centrifuge to separate and concentrate the platelets, and then injecting this enriched plasma directly into the injured area. This high concentration of platelets releases growth factors that accelerate the natural healing processes by increasing blood flow and recruiting repair cells. PRP injections can potentially reduce pain and improve joint function, making them an appealing option for patients seeking to enhance recovery in a non-surgical way. | | | | |
| **Gemini (Google Bard):**<br>PRP injections leverage your body's own healing potential. Imagine platelets in your blood as mini-toolboxes filled with growth factors. During PRP therapy, we concentrate these platelets and inject them into your injured tendon, ligament, or joint. The growth factors act like signals, recruiting nearby cells to repair and rebuild the damaged tissue. Think of it as a localized boost to your body's natural healing | | | | |

| | | | | |
|---|---|---|---|---|
| process.<br><br>It's important to understand that PRP research is ongoing, and while promising, results can vary.  We'll discuss the specifics of your case and potential benefits during a consultation. | | | | |

| **Question:**<br>*"What conditions can PRP treat?"* | excellent answer not requiring clarification | satisfactory, requiring minimal clarification | satisfactory, requiring moderate clarification | unsatisfactory, requiring substantial clarification |
|---|---|---|---|---|
| **ChatGPT-3.5:**<br>Platelet-rich plasma (PRP) shows promise in treating various orthopedic conditions, including tendon injuries (such as tennis elbow and Achilles tendonitis), ligament injuries (like ACL tears), osteoarthritis, and muscle injuries. Research suggests PRP can promote tissue healing and reduce inflammation, easing pain and improving function. However, its effectiveness can vary based on factors like the specific condition, severity, and individual response. PRP is often considered when conservative treatments fail or as an adjunct to surgery. It's crucial to consult with a healthcare professional to determine if PRP is suitable for your condition and to discuss potential benefits, risks, and alternatives comprehensively. | | | | |
| **ChatGPT-4:**<br>Platelet-rich plasma (PRP) is a treatment that uses a patient's own blood components to promote healing in various conditions. In an orthopedic and surgical setting, PRP is particularly effective in treating:<br>1. Osteoarthritis: Especially in the knee, PRP can reduce inflammation and slow the progression of arthritis.<br>2. Tendon injuries: Commonly used for conditions like tennis elbow, Achilles tendonitis, and other chronic tendon issues where it helps in healing and reducing pain.<br>3. Ligament sprains and muscle injuries: PRP can enhance the body's natural healing processes in acute sports injuries.<br>4. Post-surgical repair: It supports faster recovery after surgery for some joint, tendon, and ligament repairs by promoting tissue regeneration.<br>PRP therapy involves drawing a sample of your blood, processing it to concentrate the platelets, and then injecting it into the affected area to stimulate and enhance healing. | | | | |
| **Gemini (Google Bard):**<br>PRP therapy uses your own concentrated platelets, rich in healing factors, to jumpstart your body's natural repair process. While research is ongoing, PRP has shown promise in treating various orthopedic conditions like:<br>• Tendonitis: PRP can help heal inflamed tendons in your shoulder (rotator cuff), elbow (tennis | | | | |

| | | | | |
|---|---|---|---|---|
| elbow), knee (jumper's knee), and Achilles tendon.<br>• Ligament Sprains: PRP injections may improve healing time for ligament sprains in your ankle, knee, and other joints.<br>• Arthritis: Early studies suggest PRP may reduce pain and inflammation in osteoarthritis of the knee.<br>• Bone Injuries: PRP may aid bone healing after fractures, especially in promoting bone growth in certain cases.<br>It's important to note that PRP isn't a magic bullet, and success can vary. We'll discuss the specifics of your condition and if PRP is a suitable option for you. | | | | |

| **Question:**<br>*"How long does it take to recover from a PRP therapy?"* | excellent answer not requiring clarification | satisfactory, requiring minimal clarification | satisfactory, requiring moderate clarification | unsatisfactory, requiring substantial clarification |
|---|---|---|---|---|
| **ChatGPT-3.5:**<br>Recovery from platelet-rich plasma (PRP) therapy varies depending on the individual and the condition being treated. Typically, patients may experience initial soreness or mild discomfort at the injection site for a few days. However, full recovery can take several weeks to months as the body undergoes its healing process. Patients are advised to follow post-procedure guidelines provided by their healthcare provider, which may include rest, ice, gentle exercise, and avoiding activities that could strain the treated area. It's essential to understand that PRP therapy is a gradual process, and results may not be immediate. Regular follow-up appointments will allow your healthcare provider to monitor your progress and adjust your treatment plan as needed. Always consult your healthcare provider for personalized advice regarding your recovery timeline. | | | | |
| **ChatGPT-4:**<br>As an expert in the use of platelet-rich plasma (PRP) in orthopedic settings, I can provide you with the essential information regarding recovery from PRP therapy. Generally, the recovery period after PRP therapy can vary depending on the specific condition being treated and the individual's overall health. However, most patients begin to notice improvement in symptoms within a few weeks. The full benefits of PRP therapy typically emerge after several months. It's important to note that PRP is a minimally invasive procedure, so there's usually no significant downtime—you can often return to daily activities almost immediately. Your doctor will provide specific guidance based on your treatment plan, including any necessary activity modifications or follow-up treatments to optimize healing. | | | | |
| **Gemini (Google Bard):**<br>PRP recovery depends on the treated area. In general, expect some soreness for a few days, especially after joint injections. Most patients feel better within 2 weeks, but it can take 4-6 weeks for full recovery. Musculoskeletal injuries might require longer, up to 3-6 months.<br><br>The key is to listen to your body and avoid strenuous activity for a while. Physical therapy can also be helpful. We'll discuss a personalized recovery plan at your follow-up appointment. Remember, PRP helps your body heal itself, and that takes time. | | | | |

| **Question:**<br>*"How often should I repeat platelet-rich plasma procedures?"* | excellent answer not requiring clarification | satisfactory, requiring minimal clarification | satisfactory, requiring moderate clarification | unsatisfactory, requiring substantial clarification |
|---|---|---|---|---|
| **ChatGPT-3.5:**<br>In orthopedic and surgical settings, the frequency of platelet-rich plasma (PRP) procedures varies based on individual needs and treatment response. Typically, a series of treatments spaced several weeks apart is recommended initially, followed by periodic maintenance sessions as needed. Your response to PRP therapy, along with the severity and chronicity of your condition, will guide the frequency. Regular follow-ups with your healthcare provider are crucial to assess progress and adjust the treatment plan accordingly. Factors such as age, overall health, and activity level also influence treatment intervals. Ultimately, personalized consultation with your orthopedic specialist will determine the optimal frequency for PRP procedures tailored to your specific situation and treatment goals. | | | | |
| **ChatGPT-4:**<br>The frequency of platelet-rich plasma (PRP) treatments in an orthopedic or surgical setting varies based on the specific condition being treated and individual patient response. Typically, for conditions like osteoarthritis or tendinitis, PRP injections might be administered every 6 to 12 months. For acute injuries, such as ligament or muscle injuries, treatments could be more frequent initially—possibly several injections over a few weeks to months. It's essential to monitor your response to treatment and adjust accordingly. This process should be guided by a combination of your symptoms, the advice of your healthcare provider, and possibly imaging studies, to tailor the best treatment schedule for your particular needs. Always consult with a healthcare professional experienced in PRP therapy to determine the optimal frequency and number of treatments for your specific condition. | | | | |
| **Gemini (Google Bard):**<br>As an orthopedic surgeon with experience in PRP, there's no one-size-fits-all answer for repeat procedures. It depends on the injury, your body's response, and the initial PRP concentration. Often, one injection is enough, but some cases benefit from a second, spaced 4-6 weeks apart. We'll monitor your progress and discuss a personalized plan together. Remember, PRP is a relatively new therapy, and research is ongoing. I'll keep you updated on the latest findings to ensure the best course of treatment. | | | | |

| **Question:** | excellent | satisfactory, | satisfactory, | unsatisfactory, |
|---|---|---|---|---|

| *"How effective is PRP treatment?"* | answer not requiring clarification | requiring minimal clarification | requiring moderate clarification | requiring substantial clarification |
|---|---|---|---|---|
| **ChatGPT-3.5:**<br>PRP treatment has shown promise in promoting tissue healing and reducing pain in various orthopedic conditions like tendon injuries and osteoarthritis. Research suggests that PRP can enhance the body's natural healing processes by delivering a concentrated dose of platelets, growth factors, and cytokines to the injured area. While results vary depending on the condition and individual response, many patients experience significant improvement in pain and function. However, PRP may not be effective for everyone, and multiple treatments may be necessary for optimal results. It's essential to discuss expectations and potential risks with your healthcare provider to determine if PRP is suitable for you. Overall, PRP offers a safe and promising option for managing certain orthopedic conditions, but individual outcomes can vary. | | | | |
| **ChatGPT-4:**<br>Platelet-rich plasma (PRP) therapy is used to treat various orthopedic conditions, including tendon injuries, arthritis, and post-surgical recovery. It involves injecting a concentration of a patient's own platelets to accelerate healing. Research on PRP's effectiveness is promising but mixed. Some studies show significant improvement in pain and function, particularly in tendon injuries and mild to moderate osteoarthritis, while others find it comparable to more conventional treatments. The success of PRP may depend on factors like the specific condition treated, the area of the body, and the patient’s overall health. As with any treatment, it's important to discuss your specific situation with a healthcare provider to weigh the potential benefits and risks. Overall, PRP can be an effective option for certain orthopedic conditions, offering a potential alternative to more invasive procedures. | | | | |
| **Gemini (Google Bard):**<br>Alright, let's talk about PRP! It's a treatment where we concentrate your platelets, which are your body's natural healing helpers, into a shot. We can inject this around injuries or during surgery to potentially speed up healing.<br><br>The research on PRP is exciting, but still evolving. It shows promise for certain conditions like tendonitis or ligament tears, but results can vary.  There's no one-size-fits-all answer on effectiveness.<br><br>Here's the key takeaway: PRP might be a good option for you, but it's not a miracle cure. We'll need to discuss your specific injury and review the latest research together to see if it's a good fit for your recovery plan. | | | | |

| **Question:**<br>*"What are the side effects associated with PRP injections?"* | excellent answer not requiring clarification | satisfactory, requiring minimal clarification | satisfactory, requiring moderate clarification | unsatisfactory, requiring substantial clarification |
|---|---|---|---|---|
| **ChatGPT-3.5:**<br>Platelet-rich plasma (PRP) injections are generally safe, with minimal side effects. The most common ones include temporary pain or swelling at the injection site. Some patients might experience mild bruising, itching, or skin irritation, which usually resolves quickly. In rare cases, infection, nerve injury, or tissue damage may occur, but these are extremely uncommon when performed by experienced professionals using sterile techniques. It's crucial to discuss individual risks with your healthcare provider, especially if you have a history of bleeding disorders or allergies. Overall, PRP therapy offers a low-risk option for promoting tissue healing and reducing pain in orthopedic and surgical contexts. Your doctor will monitor you closely to ensure the best possible outcome. | | | | |
| **ChatGPT-4:**<br>Platelet-rich plasma (PRP) injections are generally considered safe, but they can have some side effects. Common ones include pain and discomfort at the injection site, which typically resolves within a few days. There might also be swelling and bruising, which are normal responses as your body reacts to the treatment. Infections are rare but can occur, as with any injection procedure. It's also possible to experience temporary increases in inflammation that may worsen symptoms before they improve. Importantly, because PRP uses your own blood, there is no risk of allergic reactions. Always ensure that the procedure is done by a qualified healthcare provider in a sterile environment to minimize risks. If you experience unusual symptoms after the procedure, such as severe pain, significant swelling, or fever, contact your doctor immediately. | | | | |
| **Gemini (Google Bard):**<br>I understand you're considering PRP injections. While it's a promising treatment, there can be some side effects. Most common are soreness, swelling, and bruising at the injection site, like a minor bump or pinch. These usually go away within a few days.<br><br>Less common risks include infection, bleeding, or nerve injury.  We take steps to minimize these, but it's important to be aware.<br><br>I'll discuss your individual risk factors and how to manage any discomfort during our consultation. Remember, PRP uses your own blood, so allergic reactions are rare. | | | | |

| | | | | |
|---|---|---|---|---|
| Let's chat more to see if PRP is the right choice for you. | | | | |

| **Question:**<br>*"How painful is a PRP injection?"* | excellent answer not requiring clarification | satisfactory, requiring minimal clarification | satisfactory, requiring moderate clarification | unsatisfactory, requiring substantial clarification |
|---|---|---|---|---|
| **ChatGPT-3.5:**<br>PRP injections are generally well-tolerated with minimal discomfort. Patients may experience mild pain or discomfort at the injection site, similar to a standard injection. However, this discomfort is usually brief and can be managed with over-the-counter pain relievers if needed. The procedure itself is quick, typically taking around 30 minutes, and performed in-office. Additionally, some clinics use numbing agents to further minimize discomfort. Overall, while individual experiences may vary, many patients find PRP injections to be relatively painless and well worth the potential benefits for orthopedic or surgical conditions. Always communicate any concerns or discomfort to your healthcare provider for appropriate management. | | | | |
| **ChatGPT-4:**<br>As an expert in the use of platelet-rich plasma (PRP) in an orthopedic setting, I can assure you that while PRP injections can cause discomfort, the pain level is generally manageable for most patients. The injection itself feels similar to a standard shot, involving a brief, sharp pain as the needle penetrates the skin and tissue. The area might feel sore for a few days post-injection. Pain varies depending on the injection site and the individual's pain tolerance. We use local anesthetics to minimize discomfort during the procedure. Afterwards, patients might experience temporary swelling and increased soreness, which is a normal part of the healing process stimulated by PRP. Over-the-counter pain relievers and ice can help manage these symptoms effectively. Overall, while not pain-free, the discomfort associated with PRP injections is usually considered worth the potential benefits in tissue healing and pain relief. | | | | |
| **Gemini (Google Bard):**<br>PRP injections themselves are usually well-tolerated. You might feel a prick from the numbing medication used first, and then possibly some pressure or pinch during the injection. Everyone's pain sensitivity varies, but it's generally not a severe procedure.<br><br>Afterwards, the injection site might be sore or inflamed for a day or two. Ice and over-the-counter pain relievers can help manage that discomfort. We can discuss pain management options in more detail at your consultation. | | | | |

| **Question:**<br>*"How much does PRP therapy cost, and is it covered by insurance?"* | excellent answer not requiring clarification | satisfactory, requiring minimal clarification | satisfactory, requiring moderate clarification | unsatisfactory, requiring substantial clarification |
|---|---|---|---|---|
| **ChatGPT-3.5:**<br>PRP therapy costs can vary based on factors like the specific treatment area, provider fees, and geographical location. On average, a single PRP injection may range from $500 to $2000. However, multiple injections may be needed for optimal results.<br><br>Insurance coverage for PRP therapy can be limited. Some insurance plans may cover PRP injections for certain orthopedic conditions, while others may consider it an elective or experimental treatment, thus not covered. It's essential to check with your insurance provider beforehand to understand your coverage. Alternatively, discuss payment options and potential financing plans with your healthcare provider. While cost is a consideration, prioritizing the effectiveness and safety of the treatment is crucial in making informed decisions about your orthopedic care. | | | | |
| **ChatGPT-4:**<br>Platelet-rich plasma (PRP) therapy typically costs between $500 to $2,500 per treatment, varying based on geographic location, provider, and specific treatment requirements. PRP therapy often requires multiple sessions, which can increase the total cost. Currently, most insurance plans do not cover PRP therapy as it is generally considered experimental and lacks sufficient standardized clinical guidelines. Therefore, patients usually need to pay out-of-pocket. It's essential for patients to discuss the potential costs and payment options with their provider before starting treatment. Additionally, I recommend checking with your insurance provider to see if there are any circumstances under which PRP therapy might be partially covered, such as part of a broader, medically necessary procedure. | | | | |
| **Gemini (Google Bard):**<br>PRP therapy costs can vary depending on your location, the treated area, and the doctor's experience. It typically ranges from $500 to $2,500 per treatment, and often multiple sessions are recommended. Insurance coverage is uncommon, but some plans might offer partial reimbursement.<br><br>Let's discuss your specific situation and explore all options to make the best decision for your care. | | | | |

| **Question:**<br>*"How do I know if PRP is the right treatment for me?"* | excellent answer not requiring clarification | satisfactory, requiring minimal clarification | satisfactory, requiring moderate clarification | unsatisfactory, requiring substantial clarification |
|---|---|---|---|---|
| **ChatGPT-3.5:**<br>PRP may be beneficial if you have musculoskeletal conditions like tendon injuries, osteoarthritis, or ligament tears. Consider PRP if conservative treatments haven't provided relief or if you prefer non-surgical options. Factors like age, overall health, and severity of your condition influence its effectiveness. Discuss with your orthopedic specialist to determine if PRP aligns with your needs and goals. They'll assess your medical history, conduct physical exams, and possibly recommend imaging tests to tailor the treatment plan to your specific situation. Remember, PRP isn't a one-size-fits-all solution, but it offers promising results for many patients seeking pain relief and improved function. | | | | |
| **ChatGPT-4:**<br>As an expert in the use of platelet-rich plasma (PRP) in orthopedic settings, I advise considering PRP if you have chronic tendon injuries, osteoarthritis, or are recovering from surgery and seek alternatives to invasive procedures or long-term medication use. PRP's efficacy varies, showing promise in enhancing healing and reducing pain by utilizing concentrations of your own platelets to stimulate natural repair processes. Key indicators that PRP might be suitable include persistent pain, limited improvement with conventional treatments, and a preference for regenerative therapies. Discuss with your healthcare provider to evaluate your specific condition, discuss potential benefits and risks, and determine if PRP aligns with your treatment goals. Optimal outcomes often depend on proper patient selection and specific injury characteristics. | | | | |
| **Gemini (Google Bard):**<br>As an orthopedic surgeon with experience in PRP, I understand you're curious if it's a good fit. PRP uses your own concentrated platelets to potentially accelerate healing.<br>Here's the key: PRP shows promise for certain conditions, like tendonitis or ligament injuries, but the research is still evolving.<br>To decide if PRP is right for you:<br>1. Consider the injury: PRP works best for specific tendon, ligament, or muscle issues.<br>2. Review treatment history: PRP is often recommended after conservative treatments like physical therapy haven't provided enough relief.<br>3. Understand the evidence: While PRP has potential, it's not a guaranteed fix. We'll discuss the research and weigh it against your needs. | | | | |

| | | | | |
|---|---|---|---|---|
| Let's schedule a consultation to discuss your specific situation and explore all your treatment options together. | | | | |
